# From synaptic guardian to neurodegenerative culprit: rewiring the amyloid-**β** feedback loop in Alzheimer’s disease

**DOI:** 10.1172/JCI200393

**Published:** 2025-12-15

**Authors:** Joachim Herz

**Affiliations:** Departments of Molecular Genetics, Neuroscience and Neurology, Center for Translational Neurodegeneration Research, University of Texas Southwestern Medical Center, Dallas, Texas, USA.

## Abstract

Studies of amyloid-β (Aβ) in Alzheimer’s disease pathology have revealed the peptide’s complex roles in synaptic function. The study by Siddu et al. in this issue clarifies the contexts in which Aβ peptides may be synaptogenic or synaptotoxic. This commentary integrates the study’s major findings with the salient findings of others that, over recent years, have redefined Aβ from a troublesome waste product into a physiological agent of the innate immune response and a modulator of synaptic homeostasis. Convergent evidence demonstrates how free, nonaggregated Aβ supports synaptic structure and activity, whereas oligomeric assemblies enact an adaptive brake on excitatory drive that can become maladaptive with age and inflammation. This redefined perspective on Aβ function emphasizes an evolutionarily conserved feedback loop linking neuronal activity, amyloid generation, and synaptic tuning that protects energy balance under stress but, when dysregulated, promotes proteostatic failure, persistent neuroinflammation, and network dysfunction characteristic of Alzheimer’s disease.

## Introduction

**Figure 1:**
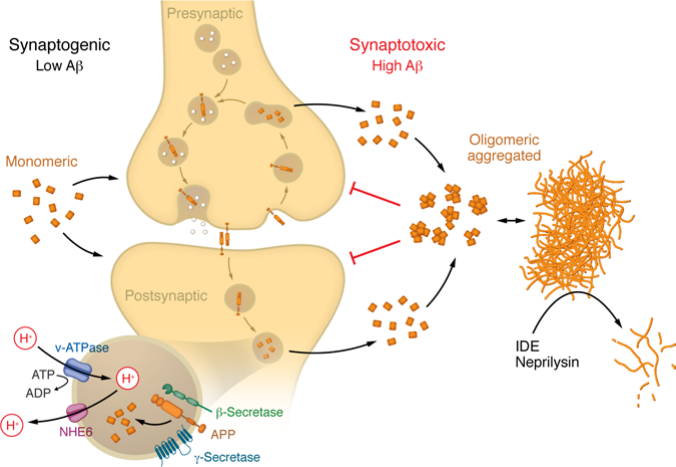
Amyloid-β aggregation state functions as a synaptic homeostatic regulator. APP is present on pre- and postsynaptic membranes, where it homo- and heterodimerizes with itself and APP-like proteins (e.g., APLP1/2) (26). Synaptic activity triggers APP endocytosis (8). The activities of the proton pump v-ATPase and the proton leak channel NHE6 determine the kinetics of endosomal acidification. The sequential action of β- and γ-secretases sets the rate of amyloid generation in pre- and postsynaptic endosomal compartments, determining extracellular Aβ concentration and aggregation kinetics. At low Aβ concentrations (left), monomers are prevalent, which stimulates synaptogenesis. As synaptic activity increases and more APP is endocytosed, or as its residence time in poorly acidified endosomes increases (16, 17), more aggregation-prone–form Aβ42 is generated, which readily oligomerizes (right). Aggregated Aβ forms interact with cell surface receptors to suppress the synapse and prevent excitotoxicity. Extracellular oligomers and aggregates are normally degraded by proteases such as insulin degrading enzyme (IDE) and neprilysin, which decrease during aging. If excessive Aβ generation persists, especially in the aging brain, Aβ oligo/polymerization outpaces the degradative capacity and persistent synaptic suppression, i.e. synaptotoxic conditions, ensue. The physiologic “sweet spot” is the point where just enough Aβ is produced to maximize synaptogenesis but not overwhelm its degradation system. This point shifts during aging due to inherently changing, environmentally and genetically determined, metabolic parameters. Therapeutic interventions can be leveraged against this shift, such as amyloid aggregate clearing antibodies (24), v-ATPase boosters such as rapamycin, or NHE inhibitors that can support an ailing proton pump and rebalance endosomal pH homeostasis (16, 17).

Alzheimer’s disease (AD) research has undergone a paradigm shift in recent years, moving from a simplistic view of amyloid-β (Aβ) as a purely neurotoxic agent to a nuanced understanding of its context-dependent roles at the synapse. The study by Siddu and colleagues (1) and related studies discussed below have been instrumental in clarifying that Aβ’s effects are determined by its aggregation state, concentration, and cellular context (Figure 1). In this brief commentary, I will attempt to synthesize selected important findings, integrating mechanistic insights from synaptic physiology, endolysosomal trafficking, and innate immunity, and discuss their implications for AD pathogenesis and therapy.

## An aggregation-dependent switch: synaptogenic versus synaptotoxic Aβ

The “amyloid hypothesis” proposed in 1991 implicated various forms of Aβ peptides in AD pathogenesis (2). Although supported by both genetic and clinical lines of evidence, the amyloid hypothesis does not explain several aspects of AD, such as the extended period between toxic Aβ production and onset of symptoms in individuals with familial AD. Moreover, clinical trial outcomes have revealed disparate outcomes for antibodies that deplete free versus aggregated Aβ peptides. In the present work, Siddu and colleagues set out to untangle the complex functional roles of Aβ in AD pathogenesis (1).

The experimental approach employed by Siddu et al. involved chronic exposure of human neurons to chemically defined synthetic Aβ species, with precise control over their aggregation state. The report focuses on species modeled on three Aβ peptides identified in humans, which are named for their amino acid (a.a.) content: the 40 a.a. peptide Aβ40, selected for its slow aggregation kinetics; the 42 a.a. Aβ42 that forms aggregates more rapidly; and an Aβ42^arctic^ variant carrying a pathogenic “arctic” mutation (E693G), which has been demonstrated to have “super aggregating” properties. Toxicity and synaptic outcomes were assessed using cell viability assays, quantitative synapse imaging, super-resolution microscopy, and calcium imaging. Sequence specificity was confirmed by showing that scrambled or inverted Aβ controls were inert, ruling out nonspecific effects. Quantitative imaging revealed that Aβ_40_ increased synapse density up to twofold, while Aβ_42_ displayed a biphasic profile: synaptogenic at low levels and synaptotoxic at higher levels. Super-resolution imaging showed a selective contraction of the presynaptic vesicle cloud at synaptotoxic Aβ_42_ levels, indicating early presynaptic failure preceding synapse elimination. Calcium imaging further demonstrated that synaptogenic Aβ increased spike frequency, while aggregated Aβ sharply reduced network activity and synchrony.

Siddu et al.’s results demonstrate that free, nonaggregated Aβ, specifically Aβ_40_ and low concentrations of Aβ_42_, acted as a synaptogenic modulator, increasing synapse density and enhancing neuronal network activity. In contrast, aggregated high-concentration Aβ_42_ and the super-aggregating Aβ_42_^Arctic^ variant were synaptotoxic and neurotoxic, disrupting presynaptic architecture and leading to synapse loss before overt neuronal death (1). This aggregation-dependent switch reconciles conflicting views in AD research, showing that Aβ’s effects are not universally harmful but depend on its physical state and concentration.

## The broader picture

Siddu et al. unify prior synaptocentric findings by showing that Aβ acts as a physiological synaptic enhancer when free, but flips to a synaptotoxic agent when aggregated. This integrates the conclusions of earlier, disconnected reports of presynaptic facilitation at low Aβ with presynaptic release suppression and postsynaptic depression caused by oligomeric Aβ. For example, Abramov et al. (3) identified endogenous Aβ as a positive regulator of release probability at hippocampal synapses, while Puzzo et al. (4) showed that picomolar Aβ_42_ enhances long-term potentiation (LTP) of synaptic strength and memory. Fogel et al. (5) linked Aβ-driven presynaptic enhancement to amyloid precursor protein (APP) homodimer signaling at boutons, providing a receptor-proximal route for physiological Aβ to tune vesicle priming.

Conversely, aggregated Aβ suppresses synaptic function. He et al. (6) demonstrated that oligomeric Aβ_42_ lowers presynaptic release probability via metabotropic glutamate receptor 5–dependent (mGluR5-dependent) phospholipase activation and depletion of the presynaptic plasma membrane phospholipid PIP2, with PIP2 replenishment rescuing transmitter release and cognition. Siddu et al. (1) observed a contraction of the presynaptic vesicle cloud under synaptotoxic conditions, consistent with these mechanistic insights. Postsynaptic suppression is also well documented: Hsieh et al. (7) showed that Aβ engages synaptic long term depression (LTD) pathways to drive endocytosis of excitatory AMPA receptors, leading to dendritic spine loss and weakened NMDA responses. Kamenetz et al. (8) found that neuronal activity–elevated Aβ depresses excitatory transmission via NMDA receptor–dependent mechanisms, embedding Aβ into a homeostatic negative feedback loop that can become maladaptive. Lauren et al. (9) implicated cellular prion protein as a cell surface receptor for oligomeric Aβ and a mediator of its suppression of plasticity, complementing postsynaptic receptor/coreceptor models relevant to oligomer-triggered LTD-like signaling pathways.

Human brain–derived Aβ oligomers provide a translational rationale for the aggregation-dependent pathological functions of Aβ: Shankar et al. (10) showed that soluble Aβ oligomers from AD cortex inhibit LTP, enhance LTD, and reduce spine density, and that, in vivo, these formed oligomers rapidly diminish synaptic efficacy and spines via NMDAR-dependent mechanisms. These functional outcomes are congruent with Siddu et al.’s aggregation-dependent switch, where oligomeric/aggregated species drive synaptotoxic collapse in contrast with the synaptogenic actions of free Aβ (1).

## Activity-dependent Aβ release and feedback control

Synaptic activity rapidly elevates extracellular Aβ, primarily through synaptic vesicle exocytosis and clathrin-mediated APP endocytosis (11). Blocking endocytosis lowers extracellular Aβ and prevents activity-driven increases, indicating that APP internalization is a dominant source of secreted peptide during heightened network activity. This forms a physiological feedback loop: increased activity leads to more Aβ secretion; higher concentrations of Aβ, especially Aβ_42_, are aggregation prone; and these oligomers/aggregates then depress synaptic transmission and curb neuronal ATP consumption, constituting a protective mechanism under stress. Kamenetz et al. (8) established that neuronal activity boosts Aβ generation and that Aβ, in turn, depresses synaptic transmission, suggesting a negative feedback loop to restrain hyperexcitability. This loop to vesicle cycling by endocytosis and exocytosis is required for activity-dependent Aβ release (11, 12), linking presynaptic trafficking to Aβ generation at active synapses. Balanced activity can support physiological Aβ signaling, whereas impaired clearance and oligomer formation shift the loop into pathology, and this is precisely the aggregation boundary Siddu et al. have experimentally defined.

## Endolysosomal acidification, proteostasis, and synaptic health

Neuroinflammation and aging disrupt endolysosomal acidification, stalling glutamate receptor recycling and impairing lysosomal degradation of Aβ and the APP β cleavage product βCTF. Im and Nixon et al. (13) proposed that when phosphorylated, βCTF binds the vaculolar (v)-ATPase subunit V0a1, impeding V1 assembly and thus reducing lysosomal acidification and hydrolase activity. This leads to increased intracellular Aβ generation and secretion, further inhibiting synaptic activities and perpetuating the pathological loop. Endosomal traffic jams represent an upstream pathogenic hub in AD (14, 15), and “unjamming” endosomes is predicted to normalize downstream proteostasis and signaling.

The AD-associated ApoE4 variant of the apolipoprotein E fat-binding protein requires a lower pH to disengage from its receptors than other variants. It exacerbates the endosomal defects that cause traffic jams by trapping ApoE and glutamate receptors in hypoacidified early endosomes (EE), preventing Reelin-driven recycling, and causing loss of surface receptors, impaired NMDA phosphorylation, and blunted LTP (14). Lowering early endosomal pH by inhibiting the proton leak channel NHE6 restores receptor recycling and synaptic plasticity (14, 16, 17). Chen et al. demonstrated that ApoE4 selectively sequestered ApoE receptor 2 and glutamate receptors intracellularly, thus suppressing Reelin signaling and its ability to maintain surface NMDA/AMPA receptors and sensitizing synapses to Aβ-mediated suppression. The reduced Reelin activity also derepressed GSK3β activity, which, in turn, favored τ hyperphosphorylation, an endpoint of the Reelin signaling pathway originally demonstrated by Hiesberger (18) and Beffert (19), and recently validated as a major AD protective mechanism in humans (20). Xian et al. (17) showed that pharmacologic or genetic NHE6 inhibition acidified early endosomes and fully reversed the ApoE4-induced recycling block, restoring Reelin modulation of excitatory synapses. Pohlkamp et al. (16) extended this by showing that NHE6 depletion corrected ApoE4-mediated synaptic impairments and reduced amyloid plaque load even in the absence of ApoE4, indicating that accelerating early endosomal acidification broadly improves proteostasis and Aβ clearance, thereby reducing the formation of synaptotoxic amyloid species.

## Amyloid-β as an antimicrobial peptide: evolutionary perspective

Aβ has been proposed to function as an antimicrobial peptide, and this original role may have provided an innate immune defense essential for the survival of early humans. Aβ binds microbial and viral glycans and oligomerizes and entraps pathogens, conferring protection in animal models and correlating with antimicrobial and antiviral activity in AD brain extracts (21). Infection can seed and accelerate β-amyloid deposition in vivo, supporting the idea that Aβ evolved as part of the innate immune system. This antimicrobial function would have been essential for the survival of early humans, providing a rapid, nonspecific defense against pathogens in the brain (22).

## From adaptive feedback to maladaptive pathology

As discussed above, activity-dependent Aβ release forms a physiological negative feedback to restrain hyperexcitability and thus prevent catastrophic neuronal ATP depletion. However, chronic Aβ accumulation, inadequate clearance, and neuroinflammation in the aging brain turn this adaptive mechanism into a maladaptive loop. Persistent antimicrobial/aggregating Aβ and microglial activation sustain sterile neuroinflammation (23), further disrupting endolysosomal acidification, increasing APP β-cleavage, and propagating autolysosome failure that fosters τ phosphorylation and aggregation. This perpetual sterile neuroinflammatory state tilts the balance towards synaptotoxicity, increased β-cleavage of APP, continuously declining endolysosomal acidification, and, ultimately, τ pathology, due to autophagosomal failure as a result of reduced acidification.

## Integrative feedback loop and pathogenic cascade

The interplay of synaptic activity, endocytosis, and Aβ release forms a tightly regulated feedback loop: (a) Enhanced activity increases APP endocytosis, increasing endosomal Aβ generation and secretion, and driving synaptic depression that curbs activity and limits excessive ATP consumption. (b) Neuroinflammation/aging is associated with reduced v-ATPase assembly via βCTF and pH dysregulation, impairing lysosomal degradation and receptor recycling, leading to more intracellular Aβ/βCTF, more secretion, and stronger synaptic suppression. (c) Aβ’s innate antimicrobial response results in aggregation and plaque seeding during insults, producing persistent sterile inflammation that entrenches endolysosomal alkalinization and autophagy failure, leading to tauopathy.

This cascade illustrates how mechanisms that evolved for acute protection and homeostatic regulation can, under chronic stress or aging, become self-perpetuating drivers of neurodegeneration.

## Therapeutic implications and strategies

Siddu et al. (1) and related studies suggest that optimal AD therapies should neutralize aggregated Aβ while preserving physiological pools of free Aβ to maintain synaptogenic support. Aggregate-preferential antibodies have shown modest cognitive benefit (24), and rising free Aβ_42_ levels in cerebrospinal fluid associate with better outcomes, whereas targeting monomers or strong suppression of BACE1 (which cleaves APP in the first step of Aβ generation) to broadly lower Aβ have underperformed or worsened cognition. The therapeutic “sweet spot” would shift the balance away from aggregates but retain soluble Aβ.

Restoring endosomal acidification, either by reducing excessive APP processing by β-secretase to reassemble v-ATPase or by partially inhibiting NHE6 to normalize receptor recycling, cargo sorting, and reacidify lysosomes, can rescue synaptic plasticity and amyloid clearance. Targeting the endosomal hub via retromer stabilization (25) may further help to reestablish proteostasis and receptor routing. Preserving the physiological, synaptogenic actions of Aβ while preventing its aggregation and downstream neurodegeneration is the key challenge. Mechanistically, this could involve four categories of approaches:

(a) Restoring acidification to reduce excessive β-CTF production and thus reassemble v-ATPase and reacidify lysosomes, improving autophagic flux and proteolysis.

(b) Reacidifying EEs by partially inhibiting NHE6 and accelerating EE maturation, normalize fast recycling of ApoE and glutamate receptors, and enhance Aβ clearance while rescuing synaptic plasticity.

(c) Unjamming trafficking to target the endosomal hub through NHE6 inhibition, retromer stabilization, and improved cargo sorting that reestablishes proteostasis and receptor routing.

(d) Preserving physiology while limiting pathology to shift Aβ toward nonaggregated, synaptogenic pools and away from oligomers/plaques, which could maintain adaptive feedback without chronic synaptotoxicity. This can be achieved with a class of small molecules called gamma-secretase modulators (GSM). Such drugs are currently undergoing evaluation in clinical trials.

## Limitations and open questions

Most experiments in the Siddu et al. (1) study were performed in human neurons cocultured on mouse glia, and the precise receptors or pathways mediating synaptogenic versus synaptotoxic Aβ actions remain to be identified. Synaptotoxicity appears more widespread than frank neurotoxicity under chronic aggregate exposure, but whether other cell types are directly affected requires further study. The interplay between antimicrobial defense, neuroinflammation, and proteostasis in the aging brain remains an active area of investigation. Further research is needed to clarify the molecular determinants of Aβ’s aggregation-dependent switch, the role of specific receptors and signaling pathways, and the potential for therapeutic interventions that restore normal amyloidogenesis and proteostasis without disrupting physiological synaptic function.

## Conclusion

The evolving understanding of Aβ’s dual roles at synapses — modulated by aggregation state, activity, and cellular context — offers a new framework for AD pathogenesis and therapy. By integrating physiological feedback, innate immunity, and endolysosomal trafficking, we are now uncovering actionable targets to restore normal amyloidogenesis and proteostasis. Future therapies should aim to preserve the beneficial, synaptogenic actions of Aβ while preventing its aggregation and the cascade of neurodegeneration that follows. This approach holds promise for breaking the pathological cycle and restoring synaptic health in the aging brain.

## Funding support

This work is the result of NIH funding, in whole or in part, and is subject to the NIH Public Access Policy. Through acceptance of this federal funding, the NIH has been given a right to make the work publicly available in PubMed Central.

- NIH (R01NS108115).
- The Alzheimer’s Association.
- The Kleberg Foundation.
- The Texas Alzheimer’s Research and Care Consortium.
- Subctontracts on NIH Awards to Reelin Therapeutics (1R43AG094356 and R43AG084450).

## Version 1 12/15/2025

Electronic publication
